# The Spread of Plague

**Published:** 1900-03-03

**Authors:** 


### THE SPREAD OF PLAGUE.

Were it not for the absorbing interest of the war in South Africa there can be but little doubt that the steady spread of plague into fresh areas would attract much more attention than it has lately seemed to do. This strange disease has fortunately so far not laid its hand upon the contending armies, and the mystery which has always attached to it, and to the curious vagaries of its distribution, is by no means lightened by the fact that instead of taking advantage of one of the admirable opportunities which man could have devised for its dissemination—instead, that is, of attacking the armies in the field and the crowded refugees who have huddled together in the towns of South Africa, plague has gone wandering abroad among the islands of the Pacific, has attacked Brazil, and has threatened the coast line of the United States. It is the mystery of the disease, the fact that we know no reason for its renewed activity during recent years, and that we are quite in the dark as to why it should spread in one direction rather than in another, that not unnaturally renders nations which lie in its track anxious as to the future. No one can then be surprised to find anxiety on the subject showing itself in the United States, a country which is threatened on both sides, from Brazil and from the Pacific. The Hawaiian Islands are especially important in this respect, owing to their considerable trade with America. Moreover, as is pointed out by the New York *Medical Record*, the condition of many of the American towns on the west coast are not such as to warrant the belief that if the plague were to obtain an entrance its extermination would be in any sense a slight affair, for there is on that side a large population of Chinese, a race peculiarly predisposed to plague infection, while the climate, the overcrowding, and the uncleanly habits of many of the aliens on that side favour diseases of this description. Although one has to confess that in many senses plague is a mysterious disease, still the history of its various outbreaks does at least give this comforting assurance, namely, that a well-clad, well-fed, well-housed, and cleanly race tends to remain untouched even when the disease is prevalent all around. In every community, however, there are sufficient people who are neither clad, nor fed, nor housed as they should be if plague is to be kept at bay, and whose cleanliness is a minus quantity.

